# Systemic Lupus Erythematosus Presenting as Hematoma of the Hand Due to Acquired Inhibitors to Factor VIII: Early and Prolonged Remission Achieved with Upfront Rituximab

**DOI:** 10.7759/cureus.4786

**Published:** 2019-05-30

**Authors:** Abdul Rehman Z Zaidi, Mohammed AlSheef, Ibraheem H Motabi, Syed Ziauddin A Zaidi, Imran K Tailor

**Affiliations:** 1 Internal Medicine, King Fahad Medical City, Riyadh, SAU; 2 Medicine, King Fahad Medical City, Riyadh, SAU; 3 Department of Adult Hematology / Blood and Marrow Transplant, Comprehensive Cancer Center, King Fahad Medical City, Riyadh, SAU

**Keywords:** factor viii, remission, antiphospholipid antibodies, bleeding, thrombosis, hematoma, acquired inhibitors, rituximab, systemic lupus erythematosus

## Abstract

Acquired hemophilia is a rare autoimmune disorder that is a result of antibodies against clotting factor VIII and it presents with excessive or prolonged bleeding, often into the muscles. Thrombotic phenomena with lupus anticoagulant are common in patients with systemic lupus erythematosus (SLE). We report a rare case of a young female with no significant past medical history presenting with hematoma of the hand who was later on found to have acquired hemophilia, SLE with antiphospholipid antibodies (APLA). She was successfully treated with upfront rituximab and prednisolone leading to early and prolonged remission. No increased incidence of infections was noted. Upfront rituximab appears to be a safe and effective option in the management of such patients when compared to use of cytotoxic agents such as cyclophosphamide; however, further data from randomized studies is needed. Neutropenia and acquired hemophilia should also be considered to be listed under hematological manifestations of SLE diagnostic criteria, as they are not uncommon in such patients.

## Introduction

Acquired hemophilia is a rare autoimmune disorder that is a result of antibodies against clotting factor VIII and it presents with excessive or prolonged bleeding, often into the muscles. Hemarthrosis is rare unlike congenital hemophilia [1].^ ^Thrombotic phenomena with lupus anticoagulant are common in patients with systemic lupus erythematosus (SLE) [2]. We report a case of a young female with no significant past history presenting with hematoma of the hand who was later on found to have acquired hemophilia, SLE with antiphospholipid antibodies (APLA). SLE presenting as acquired hemophilia is very rarely reported.

## Case presentation

A 20-year-old, never-married Saudi female presented to the emergency department with sudden onset swelling of the right hand without any trauma. She also gave history of migratory joint swelling but no morning stiffness. She also did complain of hair loss but no rash. Her mother was a known patient with thrombocytopenia for seven years. The patient used ibuprofen for the pain. Otherwise, she had no significant past medical history. Systemic examination was unremarkable apart from the swollen right hand, both palmar and dorsal aspects; however, there was no vascular compromise.

Initial investigations showed normal complete blood count except for neutropenia with an absolute neutrophils count of 1.16 x 109/L, and coagulation screen revealed an isolated prolonged PTT of 102.9 seconds. Mixing study with pooled normal plasma (1:1) revealed non correction of activated partial thromboplastin time (APTT) (91.9 seconds), even with two hour incubation. Later, lupus anticoagulant screen was found to be negative and factor VIII level was reported to be very low (0.02%), factor IX was normal (70%), and factor VIII inhibitor level was high at 22.4 Bethesda Units (BU)/ml. Anticardiolipin antibodies to immunoglobulin G, M and anti beta 2-glycoprotein I antibody were very high. Anti-nuclear antibody screen was positive (speckled pattern, less than 1/160) with positive anti double stranded DNA antibodies. C3 and C4 levels were low. HIV serology was negative.

A diagnosis of acquired hemophilia with SLE with APLA was made. Bleeding was controlled with recombinant human factor VIIa. Prednisolone 1 mg/kg, hydroxychloroquine sulphate (HCQS) were started. Cyclophosphamide was considered, but the patient was not keen on it due to potential gonadal toxicity. As the patient’s APTT was still prolonged two weeks later with mild bleeding, she was started on rituximab (monoclonal antibody to CD20) at 375 mg/m2 weekly for a total of four weeks. She achieved normalization of APTT, factor VIII levels with disappearance of inhibitor after two weeks of initiating rituximab (see Table 1). The patient continues to be in stable remission from acquired hemophilia two years since the last rituximab with no infections or any other significant toxicity.

**Table 1:** Effect of Prednisolone/Rituximab on Factor VIII and Inhibitor Level HCQS - hydroxychloroquine sulphate, APTT - activated partial thromboplastin time

Therapies	Factor VIII Inhibitor Titre (Bethesda Units)	Factor VIII Level in Percent	APTT in Seconds	Timeline
Prednisolone 1 mg/kg, Hydroxychloroquine sulphate (HCQS), Recombinant Factor VIIa	22.4	0.02	102	Day 1
Prednisolone, HCQS, Rituximab Cycle 1	16.1	0.24	73.6	Day 14
Prednisolone, HCQS, Rituximab Cycle 2	4.8	0.75	68.0	Day 21
Prednisolone HCQS, Rituximab Cycle 3	0.50	14.50	48.8	Day 28
Prednisolone HCQS, Rituximab Cycle 4	0	75	36	Day 35
Prednisolone Taper, HCQS	0	80	35.4	Day 42
Prednisolone 2.5 mg, HCQS 200 mg	0	132	39.0	Day 180
HCQS 200 mg	0	145	32	1 year
HCQS 200 mg	0	203	23.7	2 year

## Discussion

Acquired hemophilia results from antibodies that were directed against factor VIII, most commonly the C2 domain of factor VIII. The most common associated diseases are malignancy, postpartum state, and autoimmune disorders [3].

Treatment strategies include control of bleeding and elimination of inhibitor. Treatment options to control bleeding include desmopressin, factor VIII concentrates, activated prothrombin complex concentrates (aPCC), recombinant human factor VIIa and, lately, recombinant porcine factor VIII. Minor bleeding may be manageable with desmopressin. Patients with active severe bleeding with low titer inhibitor (<5 Bethesda Units) may be managed with high dose recombinant factor VIII or aPCC or recombinant human factor VIIa or recombinant porcine factor VIII. However, recombinant factor VIII is not effective in high titer inhibitor patients unlike other factors mentioned above. Elimination of inhibitor requires use of immunosuppressive agents although there is no convincing data about the superiority of each regimen over the other. Most commonly employed regimens include glucocorticoids, glucocorticoids plus cyclophosphamide, and glucocorticoids plus rituximab [4]. Using an anti-CD20 monoclonal antibody such as rituximab along with prednisolone has been found to be effective in a number of patients and is increasingly being used as first line [4, 5]. The American College of Rheumatology (ACR) and the Systemic Lupus International Collaborating Clinics (SLICC) list leucopenia, lymphopenia, and hemolytic anemia as the hematological clinical criteria to diagnose SLE, but we propose that acquired hemophilia or absolute neutropenia should also be included in the list [6, 7]. There is some emerging data to give one additional cycle of rituximab after the disappearance of the inhibitor as consolidation, although further studies are needed to confirm the same [8]. Rituximab appears to be safe and effective in achieving early and sustained remission and it appears to be a reasonable option for upfront use in acquired hemophilia; however, further studies are needed to confirm the same.

## Conclusions

We reported a rare case of a young female presenting with hematoma of the hand who was later on found to have acquired hemophilia, SLE with APLA. Acquired hemophilia masquerading as SLE is aberrantly reported. Acquired hemophilia is a medical emergency and should be treated promptly. The study aims to emphasize that SLE can present with bleeding, not only thrombosis. Also, prolonged APTT in an SLE patient does not always indicate lupus anticoagulant. As evident in our case, rituximab appears to be safe and effective in treating acquired hemophilia.

## References

[1] Tarantino MD, Cuker A, Hardesty B, Roberts JC, Sholzberg M (2017). Recombinant porcine sequence factor VIII (rpFVIII) for acquired haemophilia A: practical clinical experience of its use in seven patients. Haemophilia.

[2] Patel K, Chaney MA (2007). Chapter 89 - Hypercoagulable states: thrombosis and embolism. Complications in Anesthesia (Second Edition).

[3] Delgado J, Jimenez-Yuste V, Hernandez-Navarro F, Villar A (2003). Acquired haemophilia: review and meta-analysis focused on therapy and prognostic factors. Br J Haematol.

[4] Collins P, Baudo F, Knoebl P (2012). Immunosuppression for acquired hemophilia A: results from the European Acquired Haemophilia Registry (EACH2). Blood.

[5] Boles JC, Key NS, Kasthuri R, Ma AD (2011). Single-center experience with rituximab as first-line immunosuppression for acquired hemophilia. J Thromb Haemost.

[6] Hochberg MC (1997). Updating the American College of Rheumatology revised criteria for the classification of systemic lupus erythematosus. Arthritis Rheum.

[7] Petri M, Orbai A, Alarcón GS (2012). Derivation and validation of the Systemic Lupus International Collaborating Clinics classification criteria for systemic lupus erythematosus. Arthritis Rheum.

[8] Zaidi SZ, Iqbal S, Tailor IK (2019). Durable remissions in acquired hemophilia patients with inhibitor-titer-based tailoring of treatment strategy optimizing rituximab usage - a case series from single institution in Saudi Arabia. Blood.

